# NIA DIVISION OF EXTRAMURAL ACTIVITIES

**DOI:** 10.1093/geroni/igad104.0837

**Published:** 2023-12-21

**Authors:** Kenneth Santora

**Affiliations:** NIA, NIH, Bethesda, Maryland, United States

## Abstract

Dr. Santora will discuss the work of the NIA Division of Extramural Activities. Dr. Santora will also be available for small group discussion.

